# A Genomic Survey of Signalling in the Myxococcaceae

**DOI:** 10.3390/microorganisms8111739

**Published:** 2020-11-06

**Authors:** David E. Whitworth, Allison Zwarycz

**Affiliations:** Institute of Biological Environmental and Rural Sciences, Aberystwyth University, Aberystwyth, SY19 7DL, UK; alz11@aber.ac.uk

**Keywords:** carotenoids, comparative genomics, development, fruiting body formation, one-component systems, quorum signalling, two-component systems, myxobacteria, Myxococcales

## Abstract

As prokaryotes diverge by evolution, essential ‘core’ genes required for conserved phenotypes are preferentially retained, while inessential ‘accessory’ genes are lost or diversify. We used the recently expanded number of myxobacterial genome sequences to investigate the conservation of their signalling proteins, focusing on two sister genera (*Myxococcus* and *Corallococcus*), and on a species within each genus (*Myxococcus xanthus* and *Corallococcus exiguus*). Four new *C. exiguus* genome sequences are also described here. Despite accessory genes accounting for substantial proportions of each myxobacterial genome, signalling proteins were found to be enriched in the core genome, with two-component system genes almost exclusively so. We also investigated the conservation of signalling proteins in three myxobacterial behaviours. The linear carotenogenesis pathway was entirely conserved, with no gene gain/loss observed. However, the modular fruiting body formation network was found to be evolutionarily plastic, with dispensable components in all modules (including components required for fruiting in the model myxobacterium *M. xanthus* DK1622). Quorum signalling (QS) is thought to be absent from most myxobacteria, however, they generally appear to be able to produce CAI-I (cholerae autoinducer-1), to sense other QS molecules, and to disrupt the QS of other organisms, potentially important abilities during predation of other prokaryotes.

## 1. Introduction

During the evolution of new species from common ancestors, phenotypic differences often emerge as a result of lineage-specific changes in underlying signalling pathways and regulatory genes. It is therefore important to understand how signalling gene sets change as organisms evolve and to be able to relate those changes to formal taxonomies. Understanding the mutability of signalling gene sets can also provide us with insights into the ecology of contemporary organisms and the molecular mechanisms of their phenotypes.

The myxobacteria (order Myxococcales) are renowned for having exceptionally large numbers of signalling genes in their genomes [1,2,3]. Particularly common are serine/threonine (Ser/Thr) kinases, which regulate target proteins by reversible phosphorylation, one-component systems (OCSs), which combine a sensory domain with an ‘output’ response effector domain, and two-component systems (TCSs), which typically comprise a sensor histidine (auto)kinase (HK) which transfers phosphoryl groups to a partner response regulator (RR), sometimes via a phosphotransfer protein (P). Myxobacterial genomes also encode numerous transcription factors (TFs), including DNA-binding transcriptional regulators (TRs), alternative sigma factors and DNA-binding OCSs.

In 2015, just twelve myxobacterial genome sequences were publicly available (including three members of family Myxococcaceae, as currently defined), and analysis of those genomes confirmed that differences in TCS gene sets scaled with phylogenetic distances between strains [3]. TCS evolution was found to be dominated by gene gain/loss rather than point mutations or intra-gene insertions/deletions [3].

By September 2020, there were 375 myxobacterial genomes available, including 102 from the Myxococcaceal family. Although not as explosive, the last decade also saw an increase in the number of known Myxococcaceal species from 8 [4] to 24, with 14 of the new species identified as a result of genome-led taxonomy [5,6,7]. Family Myxococcaceae is dominated by the genera *Corallococcus* and *Myxococcus* (synonymous with *Pyxidicoccus*), which currently contain ten and twelve species, respectively [7].

The increased availability of genome sequences has allowed pan-genomic analyses of different Myxococcaceal taxa [7,8,9]. Such analyses have revealed small core genomes (the genes shared by every member of a taxon). For *Myxococcus xanthus*, only ~75% of genes in each genome belong to the core genome, dropping to just 9% of genes when comparing species within the *Myxococcus* genus [7]. This means that a large proportion of the genes in each myxobacterial genome belong to the accessory pan-genome (genes that are absent from some genomes). Indeed, 63% of the genes constituting the pan-genome of 11 *Myxococcus* spp. type strains were found to be unique to individual species, presumably having been acquired by lineage-specific duplication (with rapid divergence), or by horizontal transfer [7].

The acquisition of new genes by a genome carries with it the metabolic cost of reproducing those extra genes and consequently results in a fitness disadvantage. The selective advantage of being able to grow faster results in an evolutionary pressure for bacteria to streamline their genomes, rapidly losing genes that do not confer a selective advantage [10]. This pressure to streamline seems to be diminished in the myxobacteria as they possess unusually large genomes, with large accessory genomes [7,11]. It has been hypothesised that the slow growth exhibited by myxobacteria may result in the reduced pressure to streamline, in turn allowing accumulation of genes that might only occasionally provide a selective advantage [11].

We expected that assessing whether specific genes are part of the core or accessory genome would allow us to distinguish between genes which are functional and contribute to core behaviours/processes, and those which are dispensable: either awaiting loss from the genome, or only beneficial under limited circumstances, or in a subset of taxa. Due to their abundance, and the wealth of knowledge regarding their functioning, we particularly wanted to assess the patterns of conservation of myxobacterial signalling genes, to better understand which pathways and processes regulate core myxobacterial functions and which are unique to individual species or strains. We therefore investigated three exemplar myxobacterial signalling pathways: carotenogenesis, fruiting body development and quorum signalling.

Production of photo-protective carotenoid pigments is regulated by illumination in *M. xanthus* via the Car pathway. Exposure to light stimulates release of the alternative sigma factor CarQ, to direct transcription of *carS* [12,13,14]. CarS is an anti-repressor of the *crt* carotenoid biosynthetic genes [15,16,17]. The pathway is essentially a long linear signalling pathway, supplemented by a second repressor, which is directly inactivated by light [18].

When a population of myxobacteria is starved, it produces a multicellular fruiting body containing myxospores [19,20,21,22]. The regulators of fruiting body formation are dominated by TCS proteins, organised into largely discrete modules. Development requires the integration of signals from multiple modules, with the secondary messengers c-di-GMP (cyclic di-GMP) and (p)ppGpp, and two major intercellular signals: A-signal is a quorum signal, while C-signalling is a consequence of cell–cell contact [22,23].

Quorum signalling (QS) pathways are simple, and multiple independent pathways can be found in some organisms [24,25]. A synthase enzyme makes the QS signal (or autoinducer, AI), which is then typically transduced by either a DNA-binding transcription factor or a TCS pathway. Typical AIs include AI-I, AI-II, CAI-I and HAI-I (autoinducer-1, autoinducer-2, cholerae autoinducer-1 and harveyi autoinducer-1) [24,25]. Myxobacteria are not known to produce any AIs, however they have recently been shown to modulate myxobacterial behaviour [26].

The signalling pathways underpinning carotenoid production, fruiting body formation and QS are therefore very different, both in organisation and in the type of regulators involved, and we hypothesised that the pathway regulators would exhibit different patterns of conservation as a consequence. To that end, we surveyed the signalling proteins found in the genomes of four distinct groups of Myxococcaceae—the ten type strains of *Corallococcus* spp., the eleven type strains of *Myxococcus/Pyxidicoccus* spp., ten strains of *M. xanthus* and ten strains of *Corallococcus exiguus* (including four genomes described here for the first time). Our analysis included TCS, Ser/Thr kinases, sigma factors, OCS and other TRs. We did not include regulatory ncRNAs (non-coding RNAs) as they have been recently surveyed elsewhere [27].

Despite their large numbers, signalling proteins (particularly TCS proteins) were found to be enriched in the core Myxococcaceal genome. While the linear carotenogenesis pathway was wholly conserved, the conservation of components of the fruiting body network was highly variable. The Myxococcaceae also generally appear to be able to produce QS signals, and to sense/disrupt the QS of other organisms.

## 2. Materials and Methods

### 2.1. Genome Sequences

Four new genome sequences are reported here. Isolates AB016, AB031, AB051 and CA048 are all wild-type strains isolated from soils in Wales, UK [28]. Genomes were sequenced by MicrobesNG (Birmingham, UK) using Illumina Inc. (San Diego, CA, USA) Hi-Seq 2500 technology. Paired-end reads were quality-checked using BWA-MEM and assembled using Spades 3.7 and Kraken 2.0 [29,30,31]. Assemblies were uploaded to Genbank, wherein annotation was applied using the PGAP-4 pipeline [32].

All genome sequences and CDS (protein coding sequences) used in this study (including the four newly sequenced genomes) were subsequently downloaded from Genbank. The newly sequenced strains were identified as *C. exiguus* by calculating ANI (Average Nucleotide Identity) and dDDH (digital DNA-DNA Hybridisation) values, as described previously [9]. The four strains all gave ANI values above 95% and dDDH values above 70% when compared with the *C. exiguus* (and no other) type strain genome.

### 2.2. Identification of Regulatory Proteins

The P2RP webserver [33] was used to identify TRs and TCS proteins among the proteins encoded by each genome. Proteins are categorised into families by P2RP on the basis of their domain architecture, according to the scheme implemented in the P2CS and P2TF databases, as described by Ortet et al. [34,35]. Homologues of signalling proteins were identified in genomes using BLASTp (NCBI, Bethesda, MD, USA), with an e-value cut-off of 0.001, discarding hits with a percentage identity lower than 50% (30% if query sequences were non-myxobacterial), coverage less than 70% of query length and/or a bit-score lower than 50.

## 3. Results

### 3.1. Selection of Sets of Genomes

To investigate variations in signalling proteins within species and within genera, we selected ten or more genomes in each of the four taxa. Ten *M. xanthus* strains were selected, including *M. xanthus* DK1622, which is the single best-characterised myxobacterium. We selected the type strains of all 11 discrete species within the *Myxococcus* genus [7], and all ten type strains in its sister genus *Corallococcus* [6]. Finally, we selected ten isolates of *C. exiguus*, which is the most commonly isolated species within the *Corallococcus* genus [28]. If more than one genome assembly was available for a strain, the assembly with the smallest number of contigs was chosen.

The strains selected, their taxonomy and the characteristics of their genome sequences are presented in Table 1. As would be expected, genome metrics are more variable amongst the type strains within a genus than among strains from within a single species (with the exception of *C. exiguus* strains, which have an unusually variable number of CDS). All strains possess typical myxobacterial genomes: large (9–13.5 Mbp), with high % GC contents (69–71%).

### 3.2. MyxococcacealGenomes Encode Similar Numbers and Types of Regulatory Proteins

Regulatory proteins were then identified among the genome-encoded CDSs for each genome (Table 2, Supplementary Table S1). TCS proteins, OCSs, TRs and alternative sigma factors were identified and categorised using P2RP [33], while Ser/Thr Kinases were identified using BLASTp, queried with Pkn8 and Pkn14 from *M. xanthus* (MXAN_1710 and MXAN_5116), which both contain the pfam domain Pkinase, PF00069 [40]. To compare the variability in numbers of the different types of proteins between groups of genomes, Table 2 also presents the variability coefficient (standard deviation divided by mean) for each type of protein in each group of genomes, expressed as a percentage.

The numbers of TCS proteins and Ser/Thr kinases identified closely match those few published previously [1,2,3], with most Myxococcaceal genomes encoding around 300 TCS proteins, 100 Ser/Thr kinases and 300 transcription factors. Within each set of genomes, the numbers of each type of signalling protein are broadly similar. For instance, among the set of ten *M. xanthus* genomes, the variability coefficient was less than 10% for every type of protein except for TCS phosphotransfer proteins, which are typically present in very small numbers. For TCS and RRs, the variability coefficient was particularly low: less than 1%, compared to a variability coefficient of 1.16% for the number of CDS. A similar pattern of variability was seen within the set of ten *C. exiguus* genomes, with the number of encoded phosphotransfer proteins being highly variable, but with minimal variations in the numbers of HKs and RRs (Table 2). At a genus level, more variability was seen in the numbers of all classes of regulatory genes than when considering sets of strains within a species, with *Myxococcus/Pyxidicoccus* spp. genomes exhibiting more variability in numbers of regulatory genes than those of *Corallococcus* spp. It is also noteworthy that the average *M. xanthus* genome encodes substantially fewer regulatory proteins of every type than typical for *Myxococcus/Pyxidicoccus* spp. (Table 2), and that OCS numbers were particularly variable in each taxon.

### 3.3. Different Families of Regulators Exhibit Distinct Patterns of Conservation

**Figure 1:**
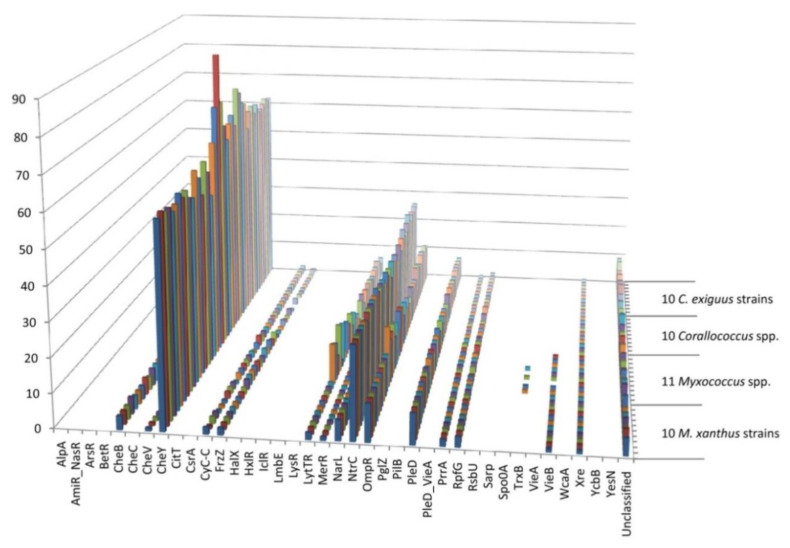
Response regulator (RR) families encoded in myxobacterial genomes. The strains under consideration are in the same order (front to back) as those detailed in Table 1 (top to bottom). Different strains and species exhibit similar profiles of RR families, although conserved differences can be seen in some groups of genomes.

TCS and TF proteins were sub-categorised into families on the basis of domain organisation according to the P2RP scheme, and the results are provided in Supplementary Table S1 [7]. Figure 1 shows the profile of RR protein families for all 41 genomes, while Table 3 provides the numbers of each family for selected protein families in each genome. The numbers of proteins in each family are broadly similar across all genomes, however, there are some consistent differences between and within groups of genomes. As noted above, for different protein classes, greater variability is observed when comparing protein families within a genus rather than within a species.

Some protein families, for example Hpt proteins, are present in small numbers, in some but not all members of a group of genomes (a single Hpt protein each is found in the genomes of just *P. fallax* and three strains of *C. exiguus*). Such proteins are not components of the core genome and are most likely to have been acquired recently by horizontal gene transfer. Other examples include the TrxB response regulator, which is found in four of the eleven *Myxococcus/Pyxidicoccus* spp. genomes, and HisKA phosphotransfer proteins, which, when present, are found in small and highly variable numbers.

Some other protein families are found in small numbers in each genome (or each genome within a group), but at a constant number. These proteins are therefore part of the core genome, and illustrative examples include CheV, HrcA, NrdR, Rok and Xre (one in each genome), VieB (one in each *Myxococcus/Pyxidicoccus* genome, but absent from *Corallococcus* genomes) and PucR (one in each *C. exiguus* genome, but only present sporadically in *Myxococcus/Pyxidicoccus* spp. and *Corallococcus* spp. genomes). Single PrrA family members are found consistently in *Corallococcus* genomes, with two members in each *Myxococcus/Pyxidicoccus* genome, Fur consistently has two members in every genome, while Cyc-C has two members per *Corallococcus* genome but one to two in *Myxococcus/Pyxidicoccus* genomes. There are two LytTR members encoded in each *M. xanthus* genome, but highly variable numbers among *Myxococcus/Pyxidicoccus* spp. members (from two to fourteen), suggesting that two of the LytTR members are part of the core *Myxococcus/Pyxidicoccus* genome, and any others are in the accessory genome.

Many protein families have larger numbers of members in each genome, and the numbers can be highly variable (for example the MerR family of TRs and OCSs has 4–6 members in each *M. xanthus* genome), or remarkably consistent (for example the OmpR family has exactly eleven members in each *M. xanthus* genome). Presumably, for each of these larger families, there will be a core set of proteins found in each genome, and a variable number of proteins from the accessory gene pool. We would therefore consider all eleven OmpR members to be core, and four MerR members to be core, with the other MerR members being part of the accessory genome.

### 3.4. TCS Proteins are More Enriched in Myxobacterial Core Genomes than Other Regulatory Proteins

To investigate the relative distribution of proteins between the core and accessory genome, we categorised the proteins in each family as ‘core’ or ‘accessory’ for each group of genomes. For this purpose, we defined the number of core proteins as simply the mean number of family members, minus one standard deviation (rounded to the closest integer), with the remainder of the proteins being categorised as members of the accessory genome. The results of such an approach are provided within Table 3 for the illustrative protein families therein, for the ten *M. xanthus* genomes. The results of this simple categorisation agree well with an intuitive assessment of core vs. accessory genome membership (Table 3).

**Figure 2:**
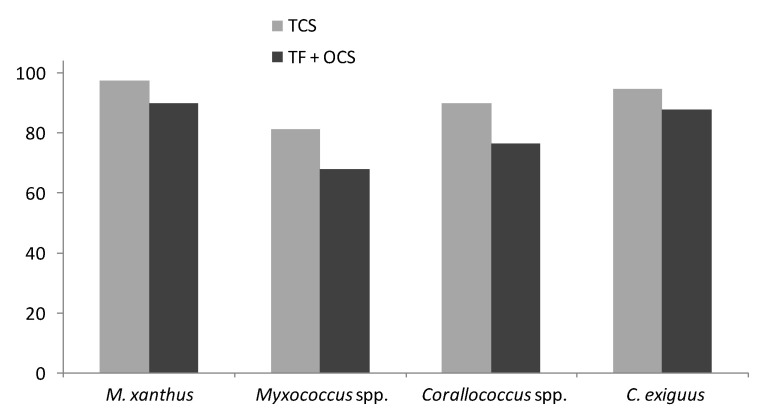
The percentage of RRs and TFs + OCSs found in the pan-genome core for the four groups of genomes.

Taking this approach, and summing the results for each protein family, we were able to compare the tendencies of RRs and TFs/OCSs to be found in the core or the accessory genome of each group of genomes (Figure 2). As expected, the percentage of proteins in the core of the pan-genome is less for *Myxococcus* spp. than for *M. xanthus* strains, as the former have more diverse genomes (similarly when comparing *Corallococcus* spp. with *C. exiguus* strains), and the *Myxococcus* spp. genomes had a smaller core than *Corallococcus* spp., reflecting their greater diversity and lower percentage core genome, as described previously [7]. Similarly, a greater proportion of *C. exiguus* regulators were found to be accessory, compared with those of *M. xanthus*, which agrees with the greater variability in the numbers of regulators in their genomes, as seen in Table 1.

In all four groups of genomes, TCS proteins were assigned to the core to a greater extent than TFs/OCSs (Figure 2), suggesting that accessory TCS proteins acquired by ‘recent’ horizontal gene transfer are purged from the genome faster than accessory TFs/OCSs. Possibly because recently acquired TCS proteins have the potential to disrupt pre-existing core TCS networks, while the expression of recently acquired TFs/OCS might be less likely to affect the functioning of core TFs/OCSs.

### 3.5. Conservation of Regulatory Proteins Involved in Key Myxococcaceal Behaviours

To further investigate the evolution of regulatory networks in Myxococcaceal genomes, we assessed the conservation of regulatory proteins in three ‘case studies’ of myxobacterial behaviours: carotenoid synthesis, fruiting body formation and quorum sensing. The regulatory mechanisms underpinning each of these phenomena are well-described and involve different classes of regulatory proteins. Identification of homologues was undertaken using BLAST, using the *M. xanthus* DK1622 protein as a query sequence (Supplementary Table S2).

**Figure 3:**
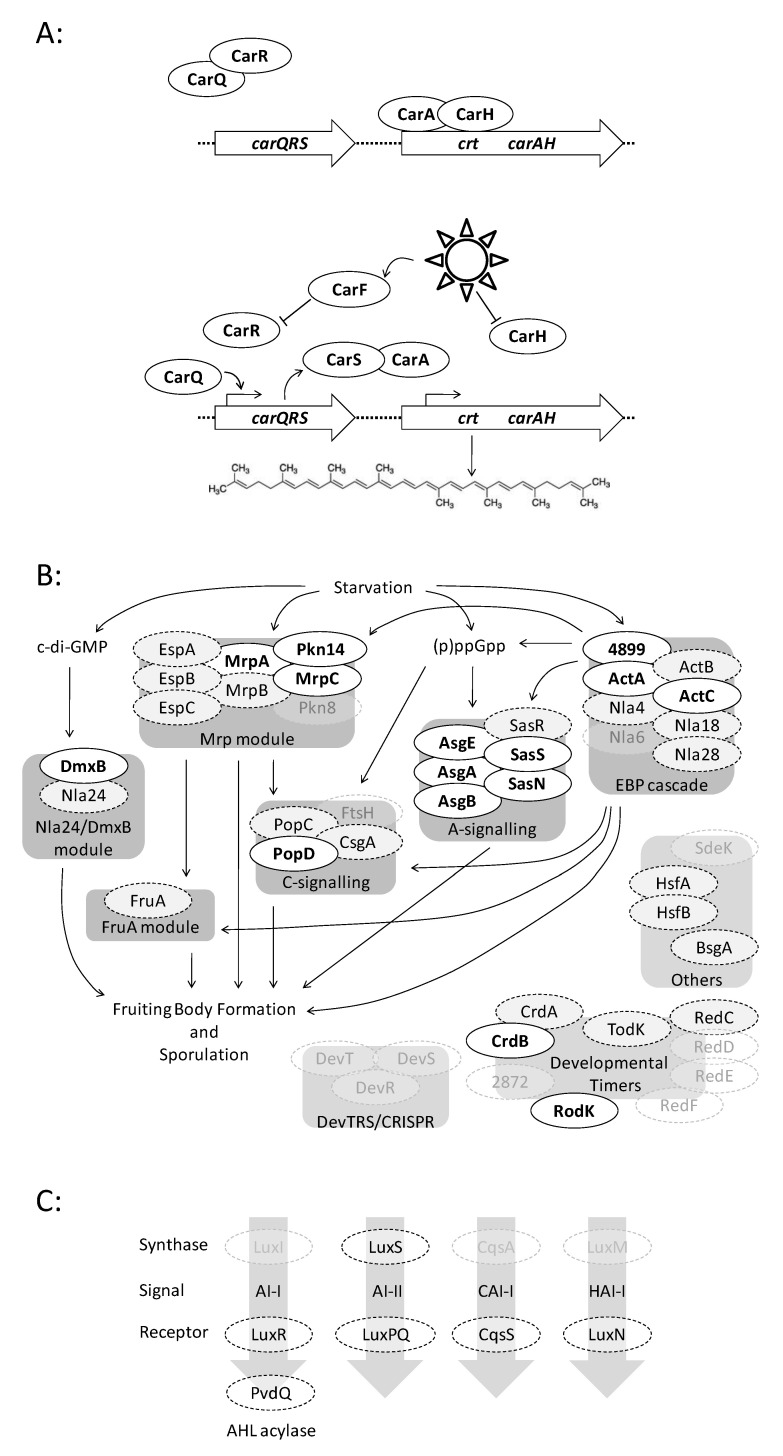
Conservation of signalling pathway proteins in Myxococcaceae. Regulatory proteins are shown as ovals. Positive regulation is shown with pointed arrows, and negative regulation with blunt-headed arrows. (**A**) Carotenoid production. In the dark (top), CarQ is held inactive by CarR, while CarA and CarH repress expression of the constitutively active *crt* promoter. In the light (bottom), CarH is directly inactivated while CarF inactivates CarR, releasing CarQ to direct transcription of *carQRS*, producing CarS which binds to CarA, relieving repression of the crt genes, which encode enzymes for the biosynthesis of carotenoids (lycopene shown as an example). (**B**) Fruiting body formation. Gene products work together in modules (dark grey boxes). Starvation triggers the production of the secondary messengers c-di-GMP and (p)ppGpp and activates the EBP (enhancer binding protein) cascade and the Mrp module. The A-signalling, C-signalling, FruA and Nla24/DmxB modules are stimulated by secondary messengers and regulatory modules. Various ‘development timer’ proteins regulate the timing of fruiting, and the DevTRS/CRISPR module modulates the timing of sporulation. ‘Other’ proteins regulate fruiting, but their relationship to other modules is not clear. (**C**) Quorum signalling. Four common Gram-negative bacterial quorum signals (AI-I, AI-II, CAI-I and HAI-I), and their corresponding synthase and receptor/regulator proteins, are shown. Also shown is the quorum-quenching AHL acylase, PvdQ. Whether regulatory proteins are core, conserved, or dispensable, is indicated based on their pattern of evolutionary conservation. Core proteins are found at a constant number per genome across the Myxococcaceae and are highlighted in bold text. Conserved proteins are found in all groups of Myxococcaceal genomes, but in variable numbers, and are indicated with a pale grey background and dashed outline. Dispensable proteins are absent from some groups of Myxococcaeal genomes and are shown with a transparent background, and grey text.

Supplementary Table S2 also shows the pattern of conservation of regulators involved in the three behaviours. Regulatory proteins were designated as ‘absent’ from a group of *n* genomes if no homologues were identified in at least *n*-1 genomes. If the same number of homologues were found in at least *n*-1 genomes, the protein was denoted ‘constant’, and if the numbers of homologues were different in at least two genomes, the protein was classified as ‘variable’. Regulators were then classified as ‘core’ (if homologues were found to be present at a constant number in all groups of genomes), ‘conserved’ (if present but found in different numbers within groups or in different groups of genomes), or ‘accessory’ (if absent from at least one group of genomes, or at least two genomes within a ‘variable’ group of genomes). Figure 3 shows the pattern of conservation of regulatory proteins involved in carotenogenesis, fruiting body formation and QS.

### 3.6. Case Study 1: Carotenogenesis

Every protein of the carotenogenesis signalling pathway was found to exhibit the same pattern of conservation (Figure 3A), with a constant single orthologue in every group of genomes (Supplementary Table S2). Thus, every component of the pathway can be considered ‘core’, and essential for the functioning of the pathway across the Myxococcaceae. This is easy to rationalise since despite integrating proteins of several regulatory classes, the pathway is essentially linear, and losing any single component results in a defective response to toxic light.

### 3.7. Case Study 2: Fruiting Body Formation

In contrast to the carotenogenesis pathway, the regulation of fruiting body formation is dominated by TCS proteins, organised into a highly interconnected network of regulatory modules (Figure 3B). The main developmental regulators were categorised into the modules or processes described by Kroos [22], with an additional category of ‘developmental timers’ as defined by Diodati et al. [41], and then homologues were identified by BLAST.

Some modules were found to be composed entirely of core/conserved gene products, for example the FruA module (one protein) and the A-signalling module (six proteins), while several modules were largely core/conserved, but included the occasional dispensable protein (Supplementary Table S2). For instance, Pkn8 appears to be dispensable from the Mrp module (eight proteins) as previously noted by Kroos [22], the EBP (enhancer binding protein) module (eight proteins) can dispense with Nla6, while the C-signalling module (four proteins) is often found without an FtsH homologue. In the two-protein Nla24 module, Nla24 is dispensable and DmxB is core (the Nla24 module should therefore be renamed the DmxB module), while developmental timers are a mixture of core/conserved (five) and dispensable (four) proteins. The DevR, DevS and DevT CRISPR (clustered regularly interspaced short palindromic repeats)-related proteins which affect the timing of sporulation were all dispensable (as noted by Kroos [22]), consistent with the proposal that they do not regulate development per se, but instead increase phage-resistance during development.

In overview, it seems that all the modules involved in regulating development are found across the Myxococcaceae, suggesting that the general organisation of the developmental pathway is evolutionarily conserved. However, the modules frequently lack proteins that are required for proper development in *M. xanthus* DK1622, implying that the developmental network is evolutionarily robust—able to evolve to cope with both the loss of developmental genes and the integration of newly acquired/duplicated gene products.

### 3.8. Case Study 3: Quorum Signalling

In contrast to carotenogenesis and fruiting body formation, QS pathways are short, and operate independently of one another. Myxobacteria are generally thought not to engage in quorum signalling, as practised by other Gram-negative bacteria, which involves the secretion of an auto-inducer signalling molecule, which producing cells then respond to. Nevertheless, using query sequences from non-myxobacterial QS organisms, homologues of various QS proteins were detected in myxobacterial genomes by BLAST (Supplementary Table S2, Figure 3C).

No genomes encoded a HAI-I synthase homologue, but an AI-I synthase was found in *C. exiguus* AB016 and an AI-II synthase was found in *M. llanfairPGensis*. Surprisingly, more than three homologues of the CAI-I synthase CqsA were encoded in each genome. The sensors of most auto-inducers are HKs, so searches for homologues of the CqsS, LuxN and LuxQ sensors produced more than 100 hits in each genome. However, homologues of the LuxR TF sensor of AI-I were less abundant but were nonetheless conserved, with at least one homologue in each genome, except that of *C. llansteffanensis*. In addition, the PvdQ AHL (acyl homoserine lactone) acylase which quenches QS had conserved homologues in every genome. Thus, it seems that production of CAI-I is a common feature of these organisms, and occasional strains can produce additional QS molecules. The capacity to sense QS molecules is conserved, including in non-producing strains (eavesdroppers), as is the ability to quench the QS of other (potential prey) organisms.

## 4. Discussion

Myxobacterial genomes encode large numbers of signalling proteins; however, within a genus, they also have very small core genomes due to the large proportion of accessory genes in each genome [7,8,9]. Previous analysis of conservation of myxobacterial TCS genes suggested that gene gain/loss was one of the most frequent types of mutational events experienced by TCS genes [3]. Nevertheless, we would expect that some TCS genes belong to the core genome and are indispensable, while other TCS genes would belong to the accessory pan-genome and would be absent from some organisms. We therefore investigated the conservation of regulatory gene family members within groups of myxobacterial genomes, and also assessed conservation of regulators associated with key myxobacterial behaviours.

The numbers of regulatory proteins of different families/classes is remarkably constant between genomes within a group of related organisms, suggesting that they are disproportionately represented in the core genome compared to ‘typical’ genes (Figure 2). TCS genes seem to be even more enriched in the core genome compared to OCSs and TFs, which perhaps reflects the large numbers of TCSs in myxobacterial genomes. Because of their shared domain architectures and mechanisms of phosphotransfer, multiple TCS signalling pathways can be integrated into sophisticated regulatory modules and networks [42]. Potentially, this might reduce the loss of individual TCS genes from genomes, with selection instead acting at the level of the whole network or module.

Fruiting body formation in *M. xanthus* is regulated by a modular network dominated by TCS proteins. However, selection does not seem to be at the level of the module. The only module that is either present or absent from different genomes in its entirety is the DevTRS/CRISPR module (Supplementary Table S2), which is thought to primarily resist phage infection during sporulation, with only secondary effects on the timing of sporulation [22]. The other regulatory modules are always present in a genome, but in every case, some individual components are conserved, while others are dispensable (Figure 3).

Robustness is a global property of modular biological networks, and the lack of conservation of ‘key’ fruiting regulatory proteins in species/strains which are proficient in fruiting implies the myxobacterial developmental network is evolutionarily robust. The impact of mutational loss can be reduced by the architecture of signalling networks [43]. It has long been recognised that robustness is an emergent property of certain network architectures. In particular, modularity is an organising principle allowing the evolution of both robustness and computational complexity in gene regulatory networks [44,45].

The ease with which suppressor and bypass mutations of developmental gene mutants can be isolated supports the notion that the developmental network is evolutionarily robust. Examples abound, but one good example involves the non-coding RNA Pxr, which inhibits the initiation of fruiting body development in the presence of nutrients [46]. In one study, a mutant strain unable to relieve Pxr inhibition in response to starvation could be restored to developmental proficiency by mutations within three separate genes, *pxr* and two positive regulators of *pxr* expression, leading the authors to conclude that reversion of developmental defects could be commonplace [47]. In another example, a third separate bypass suppressor mutation of the protease gene *bsgA* was mapped to an operon encoding RNase D and an aminopeptidase [48].

The network must also be able to incorporate newly acquired or duplicated genes. Potentially, subtle changes in phenotype due to acquisition of a new gene might confer enough of a selective advantage to promote retention of that gene. Gene duplication seems to have contributed to the large expansion in the size of myxobacterial genomes compared to the other Deltaproteobacterial Orders, with EBPs and TCSs notably prevalent [36], while acquisition by horizontal transfer might explain the origin of more than 20% of contemporary myxobacterial genes [49]. It is possible that TCSs are particularly abundant in the fruiting regulatory network because they are better able than other regulators to tolerate changes to network architecture and to engage in complex interactions with multiple partner regulators.

In contrast to the fruiting body formation network, the Car system of *M. xanthus* is essentially a linear signalling pathway, reliant on the sequential action of different categories of regulators. Unsurprisingly, all Car pathway genes are conserved in all genomes analysed (Figure 3, Supplementary Table S2). Such a pattern of conservation implies that the Car pathway regulates a phenotype with a strong selective advantage. Absence of carotenoid biosynthesis would make cells sensitive to singlet oxygen-mediated damage, resulting in death and a clear selective pressure. But, presumably, the metabolic costs of producing photoprotective carotenoids constitutively are also high enough to make retention of the signalling pathway evolutionarily favourable.

Myxobacteria are generally considered to not produce the AHLs that mediate QS in diverse Gram-negative bacteria, although recently, a cryptic myxobacterial gene resembling an AHL synthase (*agpI*) was identified in the myxobacterium *Archangium gephyra* [50]. The *agpI* gene was found to be able to induce production of AHLs in *Escherichia coli*, suggesting that AgpI may play a role in disrupting communication between prey. In addition, exogenously added AHLs have been found to promote the predatory behaviours of *M. xanthus* [26], suggesting that myxobacteria might eavesdrop on their prey. The conservation of an AHL acylase suggests that active disruption of prey AHL-mediated QS might be a common behaviour of predatory myxobacteria. Conservation of CAI-I synthase homologues suggests that myxobacteria may communicate amongst themselves via this form of QS, while the occasional strain may also be able to use alternative QS molecules (Figure 3, Supplementary Table S2). CAI-I signalling has been most commonly associated with marine bacteria, and diverse chemical variants of CAI-I have been described [25,51]. We predict that myxobacteria generally produce CAI-I variants and note that 90% of *Corallococcus* spp. type strains are predicted by antiSMASH 5 to produce homoserine lactones and/or butyrolactones, with the latter being QS molecules associated with the phylum Actinobacteria [7,52]. Further studies on QS in myxobacteria are needed to unravel what is likely to be a pervasive but idiosyncratic feature of their biology.

Clearly, different types of signalling pathways and behaviours exhibit differing patterns of gene conservation. For some pathways (e.g., the Car pathway), every component gene is highly conserved, some (e.g., fruiting body formation) are largely conserved but particular genes are dispensable, while others are present sporadically within a taxon (e.g., AI-I and AI-II synthases). As well as the structure of the pathway (modular vs. linear) and its evolutionary robustness, the pattern of conservation is also likely to be affected by the number of genetic loci over which the regulatory genes are found. For example, pathways present at single loci (e.g., QS pathways) can be acquired/lost in their entirety by single mutagenic events, whereas networks comprising large numbers of components encoded at multiple loci (e.g., fruiting body formation) are more likely to gain/lose sub-components rather than entire modules.

The availability of genome sequences means that knowledge gained by researching the molecular genetics of one model bacterium can be easily translated onto another organism by comparing their gene sets. It will be particularly interesting to extend these analyses to myxobacteria beyond the Myxococcaceae when more genomes become available. However, there are important caveats that must be appreciated when doing so, or we risk over-interpreting the significance of homologue presence/absence/variation [53], especially if using draft rather than complete genome sequences. Specifically, it seems that in myxobacteria, even if a regulatory pathway confers a selective advantage, individual genes involved in that process will likely only be evolutionarily conserved if the pathway is linear with a small-to-medium number of genes. For complex regulatory processes involving large numbers of genes (e.g., fruiting body formation), just because a gene is essential for that process in a model organism like *M. xanthus* DK1622, it cannot be assumed that it will also be required, or fulfilling the same role, in other members of that species/genus.

## Figures and Tables

**Table 1:** Genome properties of selected strains. A: *M. xanthus* strains, B: *Myxococcus* spp. type strains, C: *Corallococcus* spp. type strains, D: *C. exiguus* strains. In each section, the mean ± standard deviation (and as a percentage of the mean) is provided for the genome size, the % GC, and the number of protein-coding sequences. JGI = Joint Genome Institute.

A:								
**Genus**	**Species**	**Strain**	**Contigs**	**Size (Mbp)**	**% GC**	**CDS**	**Accession**	**Reference**
*Myxococcus*	*xanthus*	AB022	257	9.06	68.9	6995	VHLD00000000	[11]
*Myxococcus*	*xanthus*	AB024B	365	9.06	68.9	7013	SRLY00000000	[11]
*Myxococcus*	*xanthus*	AB056	230	9.11	69.1	7065	VHLB00000000	[11]
*Myxococcus*	*xanthus*	CA005	227	9.11	68.9	7175	SRLV00000000	[11]
*Myxococcus*	*xanthus*	CA006	360	9.05	68.9	6991	SRLU00000000	[11]
*Myxococcus*	*xanthus*	CA010	250	9.05	68.9	6981	VHLA00000000	[11]
*Myxococcus*	*xanthus*	CA018	727	9.07	68.8	7102	JAAEAG000000000	[9]
*Myxococcus*	*xanthus*	CA023	235	9.08	68.9	7047	JAAEAH000000000	[9]
*Myxococcus*	*xanthus*	CA027	252	9.05	68.9	7001	WBSK00000000	[9]
*Myxococcus*	*xanthus*	DK1622	1	9.14	68.9	7216	GCF_000012685	[36]
				9.08 ± 0.03	68.9 ± 0.1	7059 ± 82		
B:								
**Genus**	**Species**	**Strain**	**Contigs**	**Size (Mbp)**	**% GC**	**CDS**	**Accession**	**Reference**
*Myxococcus*	*xanthus/virescens*	DSM 2260	57	9.24	69.2	7340	FNAJ00000000	JGI
*Myxococcus*	*eversor*	AB053B	124	11.39	68.9	8751	JAAIXY01000000000	[7]
*Myxococcus*	*fulvus*	DSM 16525	42	10.82	70.0	8318	FOIB00000000	JGI
*Myxococcus*	*hansupus*	Mixupus	1	9.49	69.2	7069	GCA_000280925	[5]
*Myxococcus*	*llanfairPGensis*	AM401	1077	12.41	68.7	9508	VIFM00000000	[7]
*Myxococcus*	*macrosporus*	DSM 14697	1	8.97	70.6	6966	GCA_002305895	[37]
*Myxococcus*	*stipitatus*	DSM 14675	1	10.35	69.2	7796	GCA_000331735	[38]
*Myxococcus*	*vastator*	AM301	1008	8.99	69.9	7055	JAAIYB000000000	[7]
*Pyxidicoccus*	*caerfyrddinensis*	CA032A	177	12.67	70.2	9986	JAAIYA000000000	[7]
*Pyxidicoccus*	*fallax*	DSM 14698	825	13.53	70.5	10513	JABBJJ000000000	[7]
*Pyxidicoccus*	*trucidator*	CA060A	136	12.67	70.3	9355	JAAIXZ000000000	[7]
				10.96 ± 1.68	69.7 ± 0.6	8423 ± 1279		
C:								
**Genus**	**Species**	**Strain**	**Contigs**	**Size (Mbp)**	**% GC**	**CDS**	**Accession**	**Reference**
*Corallococcus*	*aberyswythensis*	AB050A	625	9.98	70.0	7905	RAWK00000000	[8]
*Corallococcus*	*carmarthensis*	CA043D	530	10.79	69.9	8511	RAWE00000000	[8]
*Corallococcus*	*coralloides*	DSM 2259	1	10.08	69.9	7893	GCA_000255295	[39]
*Corallococcus*	*exercitus*	AB043A	961	10.15	70.3	8018	RAVW00000000	[8]
*Corallococcus*	*interemptor*	AB047A	459	9.47	70.0	7566	RAWM00000000	[8]
*Corallococcus*	*llansteffanensis*	CA051B	1244	10.53	70.3	8137	RAWB00000000	[8]
*Corallococcus*	*praedator*	CA031B	1491	10.51	69.7	8167	RAWI00000000	[8]
*Corallococcus*	*sicarius*	CA040B	802	10.39	70.2	7877	RAWG00000000	[8]
*Corallococcus*	*terminator*	CA054A	863	10.35	69.5	8008	RAVZ00000000	[8]
*Corallococcus*	*exiguus*	DSM 14696	36	10.41	69.6	8112	JAAAPK000000000	[8]
				10.27 ± 0.37	69.9 ± 0.3	8019 ± 245		
D:								
**Genus**	**Species**	**Strain**	**Contigs**	**Size (Mbp)**	**% GC**	**CDS**	**Accession**	**Reference**
*Corallococcus*	*exiguus*	AB004	735	10.60	69.4	8223	RAWS00000000	[8]
*Corallococcus*	*exiguus*	AB016	1212	10.75	69.6	8940	JABEKY000000000	This Study
*Corallococcus*	*exiguus*	AB018	647	10.45	69.4	8185	RAWR00000000	[8]
*Corallococcus*	*exiguus*	AB030	552	10.63	69.6	8334	RAWQ00000000	[8]
*Corallococcus*	*exiguus*	AB031	611	10.43	69.7	8356	JABEKZ000000000	This Study
*Corallococcus*	*exiguus*	AB032C	298	10.45	69.5	8078	RAWP00000000	[8]
*Corallococcus*	*exiguus*	AB038B	471	10.77	69.3	8409	RAWO00000000	[8]
*Corallococcus*	*exiguus*	AB051	1378	10.76	69.6	9025	JABELA000000000	This Study
*Corallococcus*	*exiguus*	CA041A	794	10.26	69.5	8071	RAWF00000000	[8]
*Corallococcus*	*exiguus*	CA048	723	10.35	69.6	8428	JABELB000000000	This Study
				10.55 ± 0.18	69.5 ± 0.1	8405 ± 330		

**Table 2:** Variability in the numbers of regulatory genes per genome. For each class of protein, the mean number (± standard deviation (sd)), and the variability coefficient (standard deviation as a function of the mean) are presented, for four taxonomic groupings (the number of strains in each taxonomic grouping is indicated in parentheses). Variability coefficients greater than 10% are in **bold**, and values for genome-wide numbers of CDS (protein coding sequences) are provided for comparison. Gray rows represent classes, while white rows are sub-classes of the class above. TCS (two-component system) and TF (transcription factor) proteins are subdivided into different sub-classes based on domain organisation. HK = histidine kinase, P = phosphotransfer protein, RR = response regulator, TR = transcriptional regulator, OCS = one-component system.

	*M. xanthus* Strains (10)		*Myxococcus/Pyxidicoccus* spp. (11)		*Corallococcus* spp. (10)		*C. exiguus* Strains (10)	
	(mean ± sd)	(% Variability)	(mean ± sd)	(% Variability)	(mean ± sd)	(% Variability)	(mean ± sd)	(% Variability)
TCS	280 ± 2	0.81	329 ± 51	**15.58**	306 ± 12	4.05	302 ± 3	1.15
HK	141 ± 2	1.62	174 ± 35	**19.94**	160 ± 6	3.98	154 ± 2	1.00
P	3 ± 1	**22.22**	3 ± 1	**33.92**	3 ± 1	**31.43**	4 ± 2	**40.9**
RR	136 ± 1	0.90	151 ± 17	**11.13**	143 ± 7	4.56	143 ± 2	1.05
S/T Kinases	92 ± 5	5.25	124 ± 31	**25.00**	107 ± 8	7.00	113 ± 5	4.43
TF	263 ± 5	1.73	363 ± 79	**21.86**	341 ± 24	7.04	358 ± 10	2.89
TR	123 ± 2	1.33	171 ± 38	**22.04**	165 ± 11	6.92	178 ± 10	5.52
OCS	36 ± 3	7.18	70 ± 27	**38.46**	73 ± 9	**11.58**	71 ± 7	9.71
RR TF	50 ± 0	0.85	58 ± 8	**13.42**	48 ± 4	8.43	53 ± 1	1.96
σ factors	55 ± 1	1.77	65 ± 12	**19.08**	55 ± 3	5.75	56 ± 2	4.04
CDS	7059 ± 82	1.16	8423 ± 1279	**15.18**	8019 ± 245	3.06	8405 ± 330	3.93

**Table 3:** Numbers of illustrative protein family members per genome. Genomes are presented in four taxonomic groupings with the same background shade. Also shown are the inferred numbers of proteins in the core and accessory genomes of *M. xanthus* strains.

Protein Class	P	P	RR	RR	RR	RR	RR	RR	RR	RR	TR	TR	TR	TR	TR	TR
Protein Family	HisKA	Hpt	CheV	CyC-C	LytTR	OmpR	PrrA	TrxB	VieB	Xre	MerR	Fur	HrcA	NrdR	PucR	Rok
*M. xanthus* AB022	3		1	2	2	11	2		1	1	5	2	1	1		1
*M. xanthus* AB024B	3		1	1	2	11	2		1	1	5	2	1	1		1
*M. xanthus* AB056	2		1	2	2	11	2		1	1	5	2	1	1		1
*M. xanthus* CA005	4		1	2	2	11	2		1	1	4	2	1	1		1
*M. xanthus* CA006	3		1	1	2	11	2		1	1	5	2	1	1		1
*M. xanthus* CA010	3		1	1	2	11	2		1	1	5	2	1	1		1
*M. xanthus* CA018	2		1	1	2	11	2		1	1	6	2	1	1		1
*M. xanthus* CA023	3		1	2	2	11	2		1	1	5	2	1	1		1
*M. xanthus* CA027	3		1	2	2	11	2		1	1	5	2	1	1		1
*M. xanthus* DK1622	4		1	2	2	11	2		1	1	4	2	1	1		1
Core (per *M. xanthus* strain)	2	0	1	1	2	11	2	0	1	1	4	2	1	1	0	1
Accessory (per *M. xanthus* strain)	1	0	0	0.6	0	0	0	0	0	0	0.9	0	0	0	0	0
*M. xanthus (virescens)* DSM 2260	2		1	1	2	11	2		1	1	5	2	1	1		1
*M. eversor* AB053B	3		1	2	12	18	2	1	1	1	6	2	1	1	1	1
*M. fulvus* DSM 16525	3		1	2	11	13	2	1	1	1	7	3	1	1		1
*M. hansupus* Mixupus	2		1	2	6	14	2			1	4	2	1	1		1
*M. llanfairPGensis* AM401	5		1	2	14	15	2	1	1	1	7	2	1	1		1
*M. macrosporus* DSM 14697	3		1	2	2	10	2		1	1	5	2	1	1		1
*M. stipitatus* DSM 14675	3		1	2	12	13	2	1	1	1	5	2	1	1		
*M. vastator* AM301	5		1	1	2	9	2		1	1	5	2	1	1		1
*P. caerfyrddinensis* CA032A	3		1	2	10	14	2		1	1	7	2	1	1		1
*P. fallax* DSM 14698	2	1	1	3	8	14	2		1	1	5	2	1	1	1	1
*P. trucidator* CA060A	3		1	2	10	13	2			1	7	2	1	1	1	1
*C. aberyswythensis* AB050A	3		1	2	5	9	1			1	6	2	1	1	1	1
*C. carmarthensis* CA043D	3		1	2	8	12	1			1	6	2	1	1	1	1
*C. coralloides* DSM 2259	2		1	2	5	9	1			1	7	2	1	1	1	1
*C. exercitus* AB043A	3		1	2	5	11	1			1	8	2	1	1		1
*C. interemptor* AB047A	2		1	2	2	8	1			1	5	2	1	1		1
*C. llansteffanensis* CA051B	4		1	2	7	9	1			1	5	2	1	1		1
*C. praedator* CA031B	5		1	2	8	11	1			1	7	2	1	1		1
*C. sicarius* CA040B	3		1	2	4	10	1			1	8	2	1	1		1
*C. terminator* CA054A	3		1	2	7	10	1			1	6	2	1	1		1
*C. exiguus* DSM 14696	2		1	2	6	10	1			1	6	2	1		1	1
*C. exiguus* AB004	3		1	2	7	13	1			1	6	2	1	1	1	1
*C. exiguus* AB016	8		1	2	7	13	1			1	6	2	1	1	1	1
*C. exiguus* AB018	3	1	1	2	6	11	1			1	5	2	1	1	1	1
*C. exiguus* AB030	3	1	1	2	6	11	1			1	5	2	1	1	1	1
*C. exiguus* AB031	3		1	2	7	13	1			1	6	2	1	1	1	1
*C. exiguus* AB032C	3		1	2	7	13	1			1	7	2	1	1	1	1
*C. exiguus* AB038B	3		1	2	8	12	1			1	6	2	1	1	1	1
*C. exiguus* AB051	5		1	2	7	13	1			1	6	2	1	1	1	1
*C. exiguus* CA041A	2	1	1	2	5	11	1			1	6	2	1	1	1	1
*C. exiguus* CA048	3		1	2	6	11	1			1	6	2	1	1	1	1

## Supplementary Materials

The following are available online at https://www.mdpi.com/2076-2607/8/11/1739/s1, Table S1: Regulatory proteins encoded in 41 myxobacterial genomes, plus that of the *M. xanthus* type strain DSM 16526. Table S2: Pattern of conservation and the number of homologues identified in each Myxococcaceal genome when queried with regulatory proteins involved in carotenogenesis, fruiting body formation and quorum signalling.
